# Adaptive clinical trials of three PfSPZ products for development of a whole sporozoite vaccine that prevents *Plasmodium falciparum* infection, disease and transmission

**DOI:** 10.1186/1475-2875-11-S1-O48

**Published:** 2012-10-15

**Authors:** Stephen L Hoffman

**Affiliations:** 1 Sanaria Inc, Rockville, MD, USA

## 

An ideal, single stage vaccine useful for elimination of *Plasmodium falciparum* (Pf) would prevent infection at the pre-erythrocytic stage of the parasite life cycle, thereby preventing all Pf-caused disease and transmission from humans to mosquitoes. The only approach to immunization shown to consistently induce greater than 90% protection against infection and protection sustained for at least 10-28 months has been immunization by mosquito bite with whole Pf sporozoites (SPZ) of two types. The first type, radiation-attenuated PfSPZ, invade hepatoctyes and expresses new proteins, but cannot replicate. The second type fully develop in hepatocytes, producing tens of thousands of merozoites that invade erythrocytes, but are unable to fully develop within erythrocytes because they are killed by an antimalarial drug. This approach called chemoprophylaxis with sporozoites (CPS) harnesses the infectious agent’s inherent replicative properties to amplify production of protective immunogens spanning multiple developmental stages, and then eliminates the infectious agent with an anti-infective drug before the onset of disease. Sanaria was founded to develop PfSPZ vaccines. The first vaccine developed and tested was the PfSPZ Vaccine. The PfSPZ Vaccine is comprised of aseptic, purified, radiation attenuated, cryopreserved PfSPZ. It was shown to be safe and well-tolerated when administered ID or SC to 80 volunteers in the U.S., but sub-optimally immunogenic. It is now being tested in the U.S. and soon in Tanzania when administered by IV injection, since it induced high levels of PfSPZ-specific CD8+ T cells in the livers of immunized non-human primates when administered IV. A second product, PfSPZ Challenge, is comprised of non-irradiated, fully infectious PfSPZ. PfSPZ Challenge has been shown to infect 100% of volunteers after ID or IM administration by needle and syringe. It has been or will be tested for optimization of adminstration by the ID, IM, and IV routes in 2012 or early 2013 in the Netherlands, UK, Tanzania, U.S., Germany, Spain, and Kenya. A third product, PfSPZ-CVac, is comprised of PfSPZ Challenge administered to volunteers receiving chloroquine chemoprophylaxis. It will be assessed in 2012-2013 in the Netherlands, Mali, Germany and Tanzania. Assessment of these three products in synergistic, interactive and adaptive clinical trials will facilitate progress toward optimizing administration and dosage regimen of all three whole PfSPZ products, as well as those developed in the future from genetically altered parasites, thereby facilitating licensure of one or more PfSPZ-based vaccines. Progress and plans for development will be discussed.

